# Rabies Internalizes into Primary Peripheral Neurons via Clathrin Coated Pits and Requires Fusion at the Cell Body

**DOI:** 10.1371/journal.ppat.1005753

**Published:** 2016-07-27

**Authors:** Silvia Piccinotti, Sean P. J. Whelan

**Affiliations:** Department of Microbiology and Immunobiology, Program in Virology, Harvard Medical School, Boston, Massachusetts, United States of America; University of North Carolina at Chapel Hill, UNITED STATES

## Abstract

The single glycoprotein (G) of rabies virus (RABV) dictates all viral entry steps from receptor engagement to membrane fusion. To study the uptake of RABV into primary neuronal cells in culture, we generated a recombinant vesicular stomatitis virus in which the G protein was replaced with that of the neurotropic RABV CVS-11 strain (rVSV CVS G). Using microfluidic compartmentalized culture, we examined the uptake of single virions into the termini of primary neurons of the dorsal root ganglion and ventral spinal cord. By pharmacologically disrupting endocytosis at the distal neurites, we demonstrate that rVSV CVS G uptake and infection are dependent on dynamin. Imaging of single virion uptake with fluorescent endocytic markers further identifies endocytosis via clathrin-coated pits as the predominant internalization mechanism. Transmission electron micrographs also reveal the presence of viral particles in vesicular structures consistent with incompletely coated clathrin pits. This work extends our previous findings of clathrin-mediated uptake of RABV into epithelial cells to two neuronal subtypes involved in rabies infection in vivo. Chemical perturbation of endosomal acidification in the neurite or somal compartment further shows that establishment of infection requires pH-dependent fusion of virions at the cell body. These findings correlate infectivity to existing single particle evidence of long-range endosomal transport of RABV and clathrin dependent uptake at the plasma membrane.

## Author Summary

Rabies virus is the causative agent of a generally fatal and incurable disease of the central nervous system (CNS). Rabies lethality requires that the virus invade the brain, a feat accomplished by neuronal transmission from the site of infection to the CNS. Using cultures of peripheral neurons and chemicals that perturb specific cellular entry pathways we characterize the mechanism of rabies uptake. Using high resolution confocal microscopy, we visualize individual viral particles in the process of internalization and the establishment of infection by expression of a genetically encoded marker for infection. We show that clathrin-coated pits mediate internalization of the virus into endocytic vesicles that transport the virus to the cell body. We further demonstrate that release of the viral genomic core at the cell body is required to efficiently establish infection, and provide evidence that a subset of incoming virus particles fuse at non-productive sites prior to arrival at this site. This study extends the prior knowledge by identifying the entry mechanism and the site of fusion required for effective establishment of infection in neurons.

## Introduction

Rabies virus (RABV), a member of the *Rhabdoviridae* family, is a neurotropic pathogen that causes fatal encephalitis in animals and humans. The neurotropism of RABV is conferred by its single attachment and fusion glycoprotein (G) [1]. Virulence of specific RABV strains correlates with the neuroinvasiveness of their G proteins [2], such that exchange of G of an attenuated strain with that of a pathogenic strain and vice versa confers the corresponding level of pathogenicity [1,3–5]. Although differential glycosylation [6,7], dysregulation of G expression levels [8,9], and increased induction of apoptosis [8] all contribute to G-dependent attenuation of RABV strains, it is apparent that a predominant mechanism by which G modulates rabies virulence is by dictating affinity for and spread between neurons.

Following the bite of a rabid animal, peripheral neurons serve as conduits of the virus to the CNS. Although both sensory and motor neurons can be infected [10–14], retrograde transmission of RABV dictates that motor neurons serve as the primary gateway for CNS invasion [15]. The predominant route of rabies virus entry into cells appears to be clathrin-mediated endocytosis (CME) [16–19]. Electron microscopic examination of chick embryo fibroblasts [18] and hippocampal neurons [20] show the presence of virions in coated pits. The relationship of those internalization events to infection, however, is not well established and existing studies that correlate the route of entry to eventual infection are restricted to non-neuronal cells [19]. Such studies also utilize vaccine RABV strains which may behave differently than their neurotropic counterparts.

Available evidence suggests that RABV exploits existing cellular mechanisms that relay molecular signals from distal synapses to the somatodendritic compartment [21–23]. Long-range microtubule (MT) networks connect neuronal termini to the perinuclear region and mediate bidirectional axonal transport of proteins [24], mRNAs, organelles and endosomes [24–26]. Other neurotropic viruses exploit these routes to invade the CNS, but differ in directionality of transport and mode of MT engagement [27,28]. For example, polio- [29] and adeno- [30] viruses are transported within endosomes tethered to MTs via host proteins, whereas alpha herpesviruses [31] interact with cellular motors directly via capsid and tegument proteins. For RABV, single viral particles incorporating fluorescently-tagged transmembrane and RNP proteins appear to translocate intact within axons [21]. Consistent with this, receptors recruited to virions at the plasma membrane appear to remain associated during long-range axonal transport [22,23]. Collectively these studies provide evidence that rabies viruses are transported intact within endosomes, but the significance of this transport for productive infection has not been examined [21–23].

In the present study, we combine infectivity and single particle imaging approaches to study rabies internalization and fusion from the termini of neurons of the dorsal root ganglion (DRG) and motor neuron-rich ventral spinal cords (V SC) mediated by the neurotropic Challenge Virus Strain 11 (CVS) G protein. To model natural RABV infection at neuronal termini, we adapt a polydimethylsiloxane (PDMS) microfluidic culturing platform [32,33] which physically separates neuronal cell bodies from their neurites. We demonstrate that CVS G-mediated infection is reliant on dynamin-dependent uptake processes and that the predominant endocytic route is clathrin-mediated. We provide evidence that productive infection requires viral fusion at the somatodendritic compartment following long-range transport from the neuronal termini. This work extends previous findings from epithelial cells providing evidence that infection of neuronal cells by rabies virus occurs through a clathrin-dependent entry pathway with subsequent membrane fusion occurring at the cell body.

## Results

### Restricted tropism of a recombinant VSV expressing the rabies CVS glycoprotein

**Fig 1:**
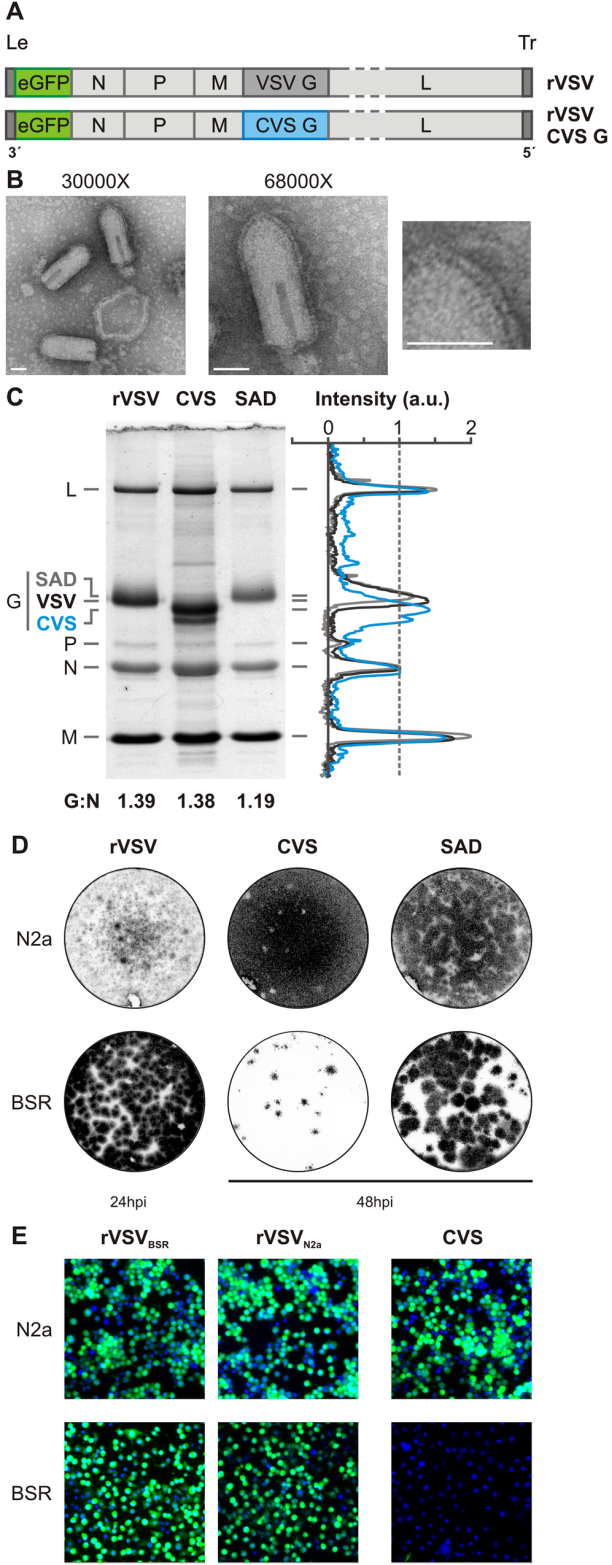
Production of a recombinant VSV expressing rabies CVS G. **A.** Organization of the negative strand RNA genomes of viruses, rVSV eGFP and rVSV eGFP CVS G. Genomes are shown in a 3′ to 5′ orientation, including the non-coding genomic leader (Le) and trailer (Tr) regions. Coding regions include the N, nucleocapsid gene; P, phosphoprotein gene; M, matrix gene; G, glycoprotein gene; L, large polymerase gene. Both viruses express an additional eGFP gene (green) as a marker for infection. In rVSV CVS G, the wild-type VSV G (1,535 nt) was replaced with the rabies G (1,575 nt) from the CVS-11 strain. **B.** Transmission electron micrographs of rVSV CVS G virions stained with 1% phosphotungstic acid. Two magnifications are included 30 000X and 68 000X. The rightmost panel is an enlargement of the area indicated and highlights the glycoprotein spikes decorating the viral surface. Scale bars = 50nm. **C.** Protein composition of purified virions by SDS-PAGE. Viral proteins from rVSV eGFP, rVSV SAD B19 G and rVSV CVS G were stained with SimplyBlue SafeStain. Intensity values normalized to N were quantified using ImageJ and are plotted for each viral lane. The calculated numerical ratio of G to N is further reported as a measure of average glycoprotein density for each virus. **D.** Fluorescent focus assay comparing infectivity of rVSV eGFP (rVSV), rVSV CVS G (CVS), and rVSV SAD B19 G (SAD) in Neuro-2A (N2a) and BSR T7/5 (BSR) cells. Fluorescent signal from eGFP expression is shown in grayscale. Infections were calibrated to obtain comparable MOIs in the N2a cells. The same infectious dose was then applied to monolayers of BSR cells for comparison. rVSV foci were evaluated at 24hpi; rVSV CVS G and rVSV SAD B19 G were evaluated at 48hpi due to slower kinetics of infection. **E.** Fluorescence microscopy of N2a and BSR monolayers inoculated with rVSV grown in BSR (rVSV_BSR_), rVSV grown in N2a (rVSV_N2a_), or CVS virus at an MOI_N2a_ of 3. Infection was assessed by eGFP expression (green) at 6hpi. Nuclei are stained with DAPI (blue).

To study uptake of RABV in neurons, we generated a recombinant VSV (rVSV) in which we replaced the glycoprotein (G) gene with that of the neurotropic rabies strain, CVS (rVSV CVS G; Fig 1A). This virus expresses eGFP as a marker of infection. Transmission electron microscopy (TEM) of purified rVSV CVS G showed characteristic bullet-shaped particles with readily discernible glycoprotein spikes (Fig 1B) consistent with efficient incorporation of CVS G. SDS-PAGE analysis of the protein composition of purified virions demonstrates comparable incorporation of CVS G into rVSV compared to RABV vaccine strain SAD B19 or VSV G (Fig 1C). CVS G migrates as a doublet consistent with the reported existence of two glycosylation variants of G [34] (Fig 1C).

Neuroinvasiveness is a defining feature of pathogenic RABV G, and incorporation of CVS G results in a corresponding shift in rVSV tropism. BSR T7/5 monolayers were less susceptible to rVSV CVS G than their non-neuroinvasive counterparts, rVSV and rVSV SAD B19 G, as evident from a reduction in the number and size of the foci (Fig 1D). In addition, rVSV CVS G displayed greater capacity for spread resulting in larger plaque sizes than its non-neurotropic counterparts in N2a cells. A viral dose equivalent to MOI = 3 in N2a cells results in a calculated effective MOI < 0.05 in BSR T7/5 assuming the standard Poisson model of infection (Fig 1E). We exclude the possibility that differential lipid composition of virus grown in N2a cells is responsible for the difference in tropism by comparing the infectivity of rVSV grown in BSR T7/5 (rVSV_BSRT7_) or N2a cells (rVSV_N2a_; Fig 1E). Cumulatively, these observations are consistent with a restricted tropism conferred by the CVS G.

### Compartmentalized culture of dorsal root ganglion and ventral spinal cord neurons

**Fig 2:**
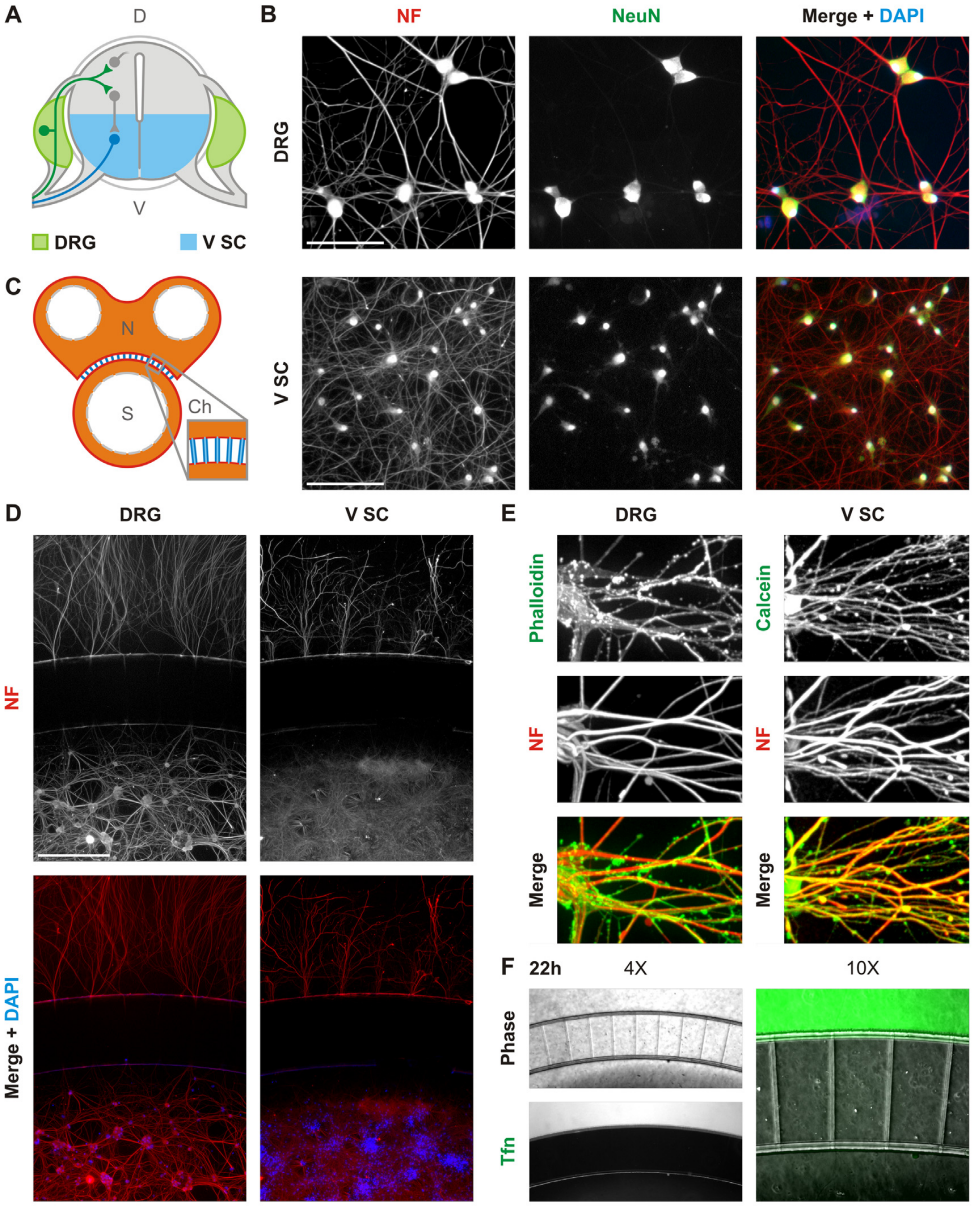
Compartmentalized cultures of dorsal root ganglion and ventral spinal cord neurons. **A.** Schematic of a cross-section of the spinal cord and dorsal root ganglia including a simplified representation of the connectivity of neurons within those tissues. Shown is a representative motor neuron cell body (blue) projecting out of the ventral (V) spinal cord (SC); a sensory neuron cell body (green) located in the dorsal root ganglion (DRG) innervates into the spinal cord; and interneurons and commissural neurons (grey) are located in the dorsal (D) spinal cord. Tissues dissected and dissociated to obtain the ventral spinal cord (V SC) and DRG neuronal cultures are indicated in blue and green respectively. Grey tissues were excluded from dissociated culture. **B.** Fluorescence microscopy of DRG and V SC dissociated culture at day 7 stained with DAPI (blue) and antibody against phosphorylated neurofilament H (NF; red) and neuronal nuclei (NeuN; green). Scale bars = 100 μm **C.** Schematic of the compartmentalized microfluidic culture device. The device includes two compartments, neurite (N) and somal (S), connected by microchannels (Ch). Color-coding indicates the depths of each device region: blue = 3 μm; orange = 100 μm; white corresponds to open wells. Microchannels are 500 μm long and 10 μm wide. **D.** Fluorescence microscopy of dissociated DRG and V SC neurons cultured in compartmentalized devices and stained against NF (red) and with DAPI (blue). Due to limited diffusion of antibodies into microchannels, neurites within these structures appear unlabeled. Scale bars = 500 μm. **E.** Confocal microscopy of fixed DRG and V SC neurites in the N compartment stained against phosphorylated neurofilament H (NF, blue), an axonal marker, by immunofluorescence. Neuronal cytoplasms were further stained with AF488-phalloidin (green) for DRG neurons and calcein (green) for V SC neurons. **F.** Abrogated diffusion of fluorescent transferrin (Tfn, green) across microfluidic channels at 22hpi.

We next investigated RABV uptake in neurons of the dorsal root ganglia (DRG) and ventral spinal cord (V SC) that project into muscle tissue (Fig 2A). Dissociated cultures, obtained from embryonic rats, yield neuronal populations with extensive projections as determined by immunofluorescence against the phosphorylated neurofilament H (NF) and the neuronal marker, Neuronal Nuclei (NeuN; Fig 2B). We selectively manipulated neuronal termini by culturing neurons in the S compartments of microfluidic devices, which allow spatial isolation of neurites from cell bodies (Fig 2C). By 10 days *in vitro* we detect significant outgrowth of projections into the distal, neurite (N) compartment (Fig 2D), and calcein staining demonstrates that neurites are contiguous across the channel (S1 Fig). Furthermore, we demonstrate by staining against phosphorylated NF that the majority of projections into the N compartment are axonal (Fig 2E). We restrict diffusion of molecules between the N and S compartments by controlling liquid levels and, therefore, hydrostatic pressure across the microchannels. Consistent with effective fluidic isolation, transferrin (Tfn) tagged with AlexaFluor 488 added to the N compartment is retained with no detectable diffusion across the channels (Fig 2F).

### Rabies G-mediated infection of neurons is dependent on dynamin

**Fig 3:**
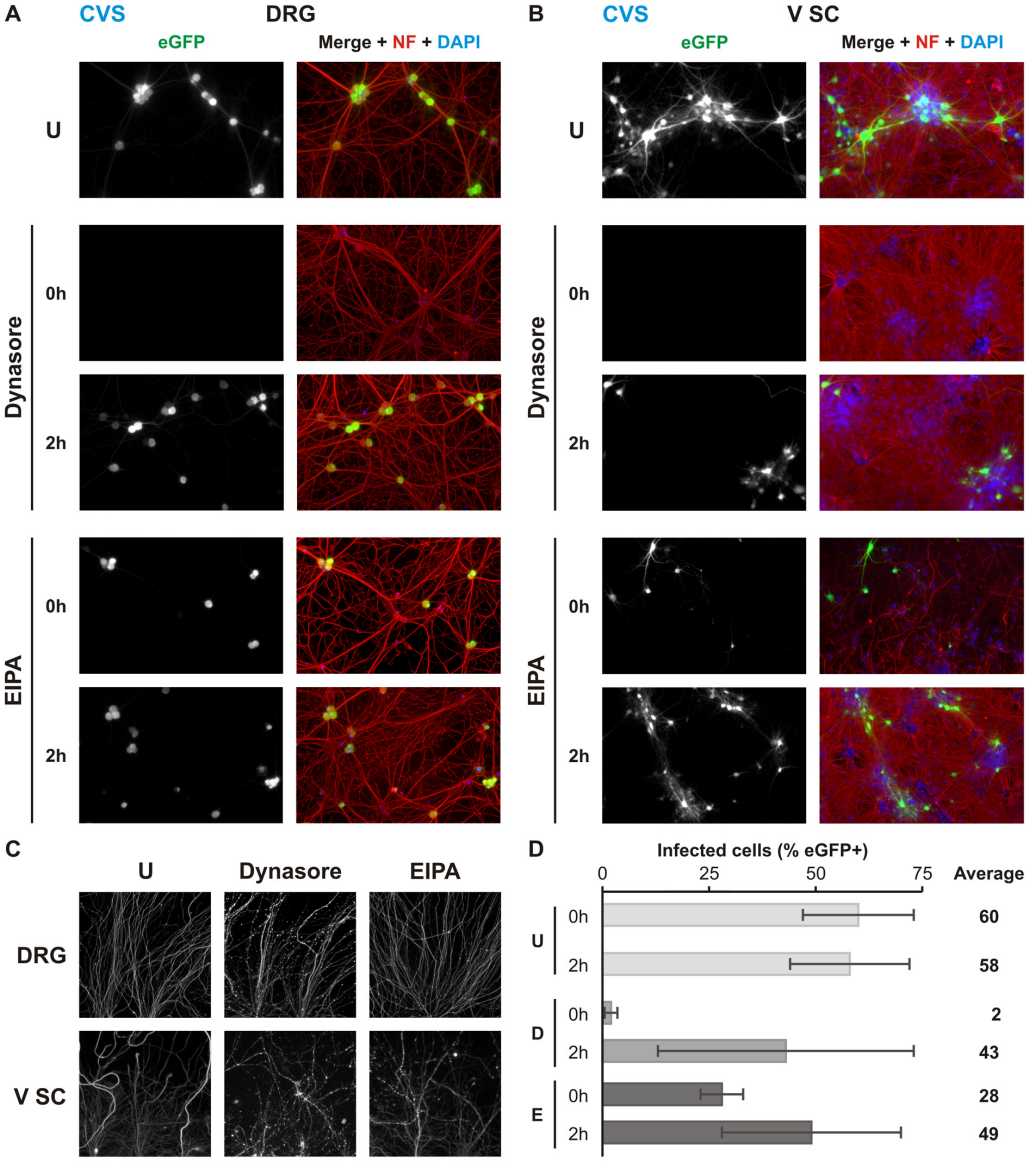
Dynasore and EIPA inhibit rVSV CVS G infection in compartmentalized neuronal culture. Fluorescence microscopy of MF cultures of **A.** DRG neurons or **B.** V SC neurons infected with rVSV CVS G in the N compartment. Neurons were additionally treated with the dynamin inhibitor, dynasore (150 μM), or the macropinocytosis inhibitor, EIPA (25 μM), as indicated at either 0 or 2 hpi and monitored for viral eGFP (green) expression at 26 hpi. Cells are further stained against phosphorylated neurofilament H (NF; red) and with DAPI (blue). **C.** Neurites in the N compartment at 26hpi and continuous treatment with the indicated inhibitor, stained against neurofilament H. **D.** Quantitation of percentage eGFP positive neurons in three iterations of the experiment in DRG culture. Error bars indicate the standard deviation for each condition.

We next evaluated the effect of endocytosis inhibitors on viral infection from the neuronal termini of cultured DRG and V SC neurons (Fig 3). For this purpose, we use dynasore and 5-(N-ethyl-N-isopropyl)amiloride (EIPA) to determine the requirement for dynamin- and macropinosome-mediated uptake respectively, and measure eGFP expression as a marker of viral infection. Consistent with dynamin-dependent endocytosis of rVSV CVS G, dynasore results in a near total block of infection in either neuronal population (Fig 3A and 3B). Treatment with EIPA also results in a reduction of infection; however, inhibition was not as pronounced as with dynasore and not significant in DRG neurons. Because neurite projections cannot be assigned to specific neurons in the somal chamber, the magnitude of the inhibitory effects of dynasore and EIPA cannot be precisely quantified. This is particularly apparent for V SC cultures which exhibit limited N compartment projection relative to the number of cultured neurons. For DRG cultures which exhibit a greater efficiency of neurite outgrowth (Fig 3C), dynasore reduces the percentage infected neurons from 43% to 2%. The inhibitory effect of EIPA in DRG infection was approximately two-fold with significant variability across experiments. This differed qualitatively from the more pronounced and reproducible inhibition observed in V SC cultures (Fig 3B). Reductions in infection by either inhibitor were not due to off-target effects on viral replication since addition of inhibitor at 2 hpi had limited effect on infection. We also exclude chemical degradation of projecting neurites as a contributing factor: intact neurite structures were retained at the experimental endpoint in both treated and untreated cultures (Fig 3D).

**Fig 4:**
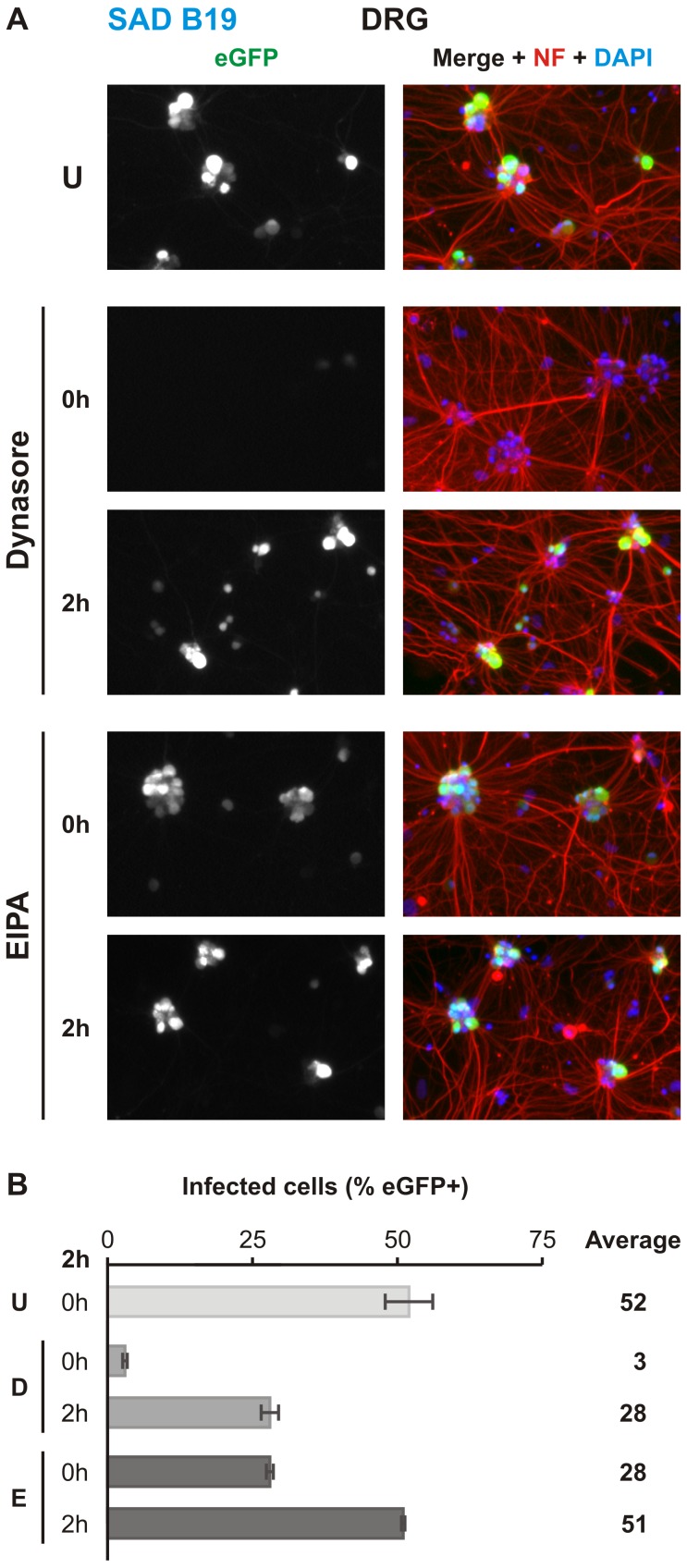
rVSV SAD B19 G infection of compartmentalized DRG neurons is dynamin-dependent. **A.** MF cultures of DRG neurons infected with rVSV SAD B19 G in the N compartment and treated with indicated inhibitor at 0 or 2hpi, also as indicated. Viral eGFP (green) expression was assessed at 26hpi. Cells are further stained against phosphorylated neurofilament H (NF; red) and with DAPI (blue). **B.** Quantitation of percentage eGFP positive neurons in three iterations of the experiment. Error bars indicate the standard deviation for each condition. Abbreviations are as follows: U, untreated; D, dynasore; E, EIPA.

We previously demonstrated that epithelial uptake of rVSV incorporating G from a vaccine strain of rabies (rVSV SAD B19 G) is clathrin-dependent [19]. To identify differences in uptake between epithelial and neuronal cells, we tested the effect of dynasore and EIPA on rVSV SAD B19 G uptake into DRG neurons (Fig 4). Treatment with dynasore resulted in an almost complete block of infection whereas EIPA treatment resulted in a more modest reduction (Fig 4B). These results suggest a shared mechanism for initial uptake at the plasma membrane during SAD B19 and CSV G-mediated entry.

### Dynamin inhibition blocks virion accumulation in the S compartment and microchannels

**Fig 5:**
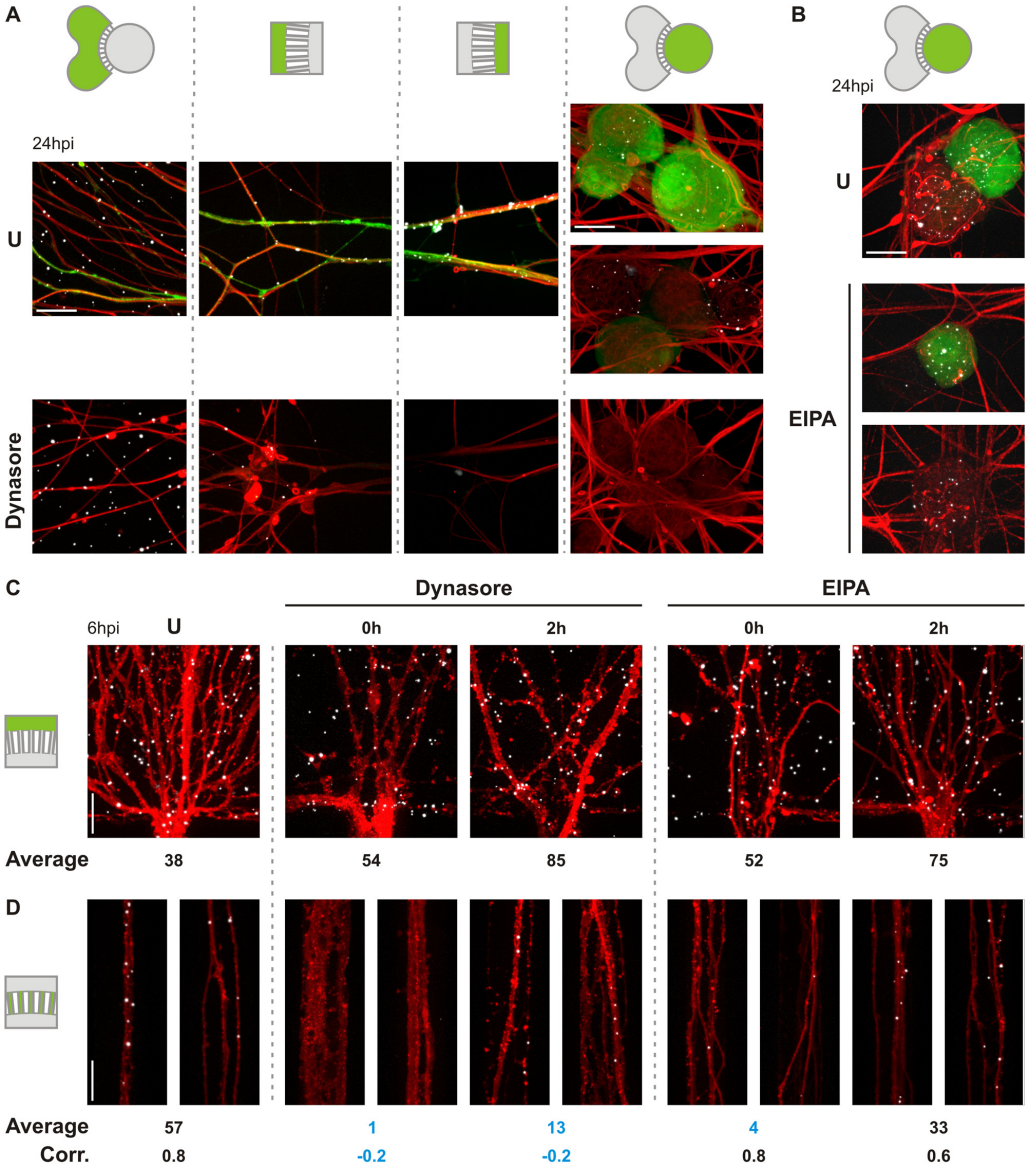
Disruption of dynamin blocks viral accumulation in microchannel neurites and cell bodies of DRG neurons in compartmentalized culture. **A.** Confocal microscopy of the localization of fluorescently-labeled rVSV CVS G particles in neurons infected in the absence (U) or presence of 150 μM dynasore at inoculation. Virus was administered to the N compartment, and the distribution of virions was assessed following reporter eGFP gene expression (green) at 24 hpi. Virus particle localization was monitored in the following structures from left to right: distal neurites, N compartment microchannel opening, S compartment microchannel opening, and S compartment cell bodies. Green highlight in device schematic indicates the compartment being imaged. The order of the panels reflects the direction of transport undergone by the virus. Neurites were stained against phosphorylated neurofilament (red). rVSV CVS G AF647 particles detected by direct fluorescence are displayed in white for ease of viewing. Scale bars = 20 μm. **B.** Confocal microscopy of rVSV CVS G AF647 (white) accumulation in the cell bodies of DRG neurons infected in the absence (U) or presence of 25 μM EIPA at inoculation. Here, infection was carried out in the N compartment and viral distribution assessed at 24 hpi in the somal compartment only. Neurons were stained against phosphorylated neurofilament (red); eGFP (green) and rVSV CVS G AF647 (white) were detected by direct fluorescence. Scale bars = 20 μm. **C. and D.** Localization of incoming rVSV CVS G AF647 particles C. along neurites at the N compartment outlet and D. within microchannels as detected by confocal microscopy at 6 hpi. Neurites were stained against phosphorylated neurofilament (red) and rVSV CVS G AF647 particles (white) were detected by direct fluorescence. N compartments of DRGs neuronal devices were inoculated with virus concomitantly (0 hpi) or 2 h prior to treatment with inhibitor as shown. U indicates untreated controls. Average number of particles per equivalent field of view is reported beneath each panel. The calculated correlation (Corr.) between particle number at the outlet and within individual microchannels is also reported for each condition. Averages and correlations that differ substantially from untreated controls are in blue for emphasis. Scale bars = 20 μm.

To determine whether chemical inhibition of infection was due to a reduction in the number of virions delivered to the soma, we next examined the cellular distribution of rVSV CVS G virions labeled with AF647 (rVSV CVS G AF647) by confocal microscopy (Fig 5). By 26 hpi neurons (Fig 5A and 5B) demonstrate efficient uptake and transport of virus: cell-associated particles are readily detected in all compartments including the cell bodies of some uninfected cells. In contrast, dynasore treatment restricts viral localization to the N compartment (Fig 5A) whereas EIPA has a limited effect on virus transport to the soma with viral particles present in both eGFP-positive and -negative neurons (Fig 5B). Irrespective of the inhibitor used, viruses efficiently bound N compartment neurites indicating that association with the plasma membrane is unaffected.

At 26 hpi, remaining intact viral particles represent a population that did not contribute to infection. We, therefore, examined rVSV CVS G AF647 uptake disruption at an earlier timepoint, 6 hpi (Fig 5C and 5D). Here, we did not detect chemical disruption of viral association with the neuronal membrane (Fig 5C) but find differential viral accumulation within microchannel neurites. Since extracellular diffusion into the microchannels is restricted, viruses must access this compartment only via axoplasmic transport and are therefore intracellular. Both dynasore and EIPA administered at the time of inoculation abrogate viral accumulation in the microchannels (Fig 5D). Dynasore also impacts viral accumulation when added at 2 hpi, although the effect was less pronounced. A strong positive correlation between the number of cell-associated viruses at the opening to and the number within individual microchannels is observed in untreated controls. Dynasore decouples N-compartment and microchannel accumulation of virus consistent with a block to particle uptake. By contrast, following EIPA treatment that correlation remains positive suggesting that EIPA delays, rather than blocks, endocytosis and transport of virus.

### Fluorescently-labeled rVSV CVS G and transferrin are transported in tandem

**Fig 6:**
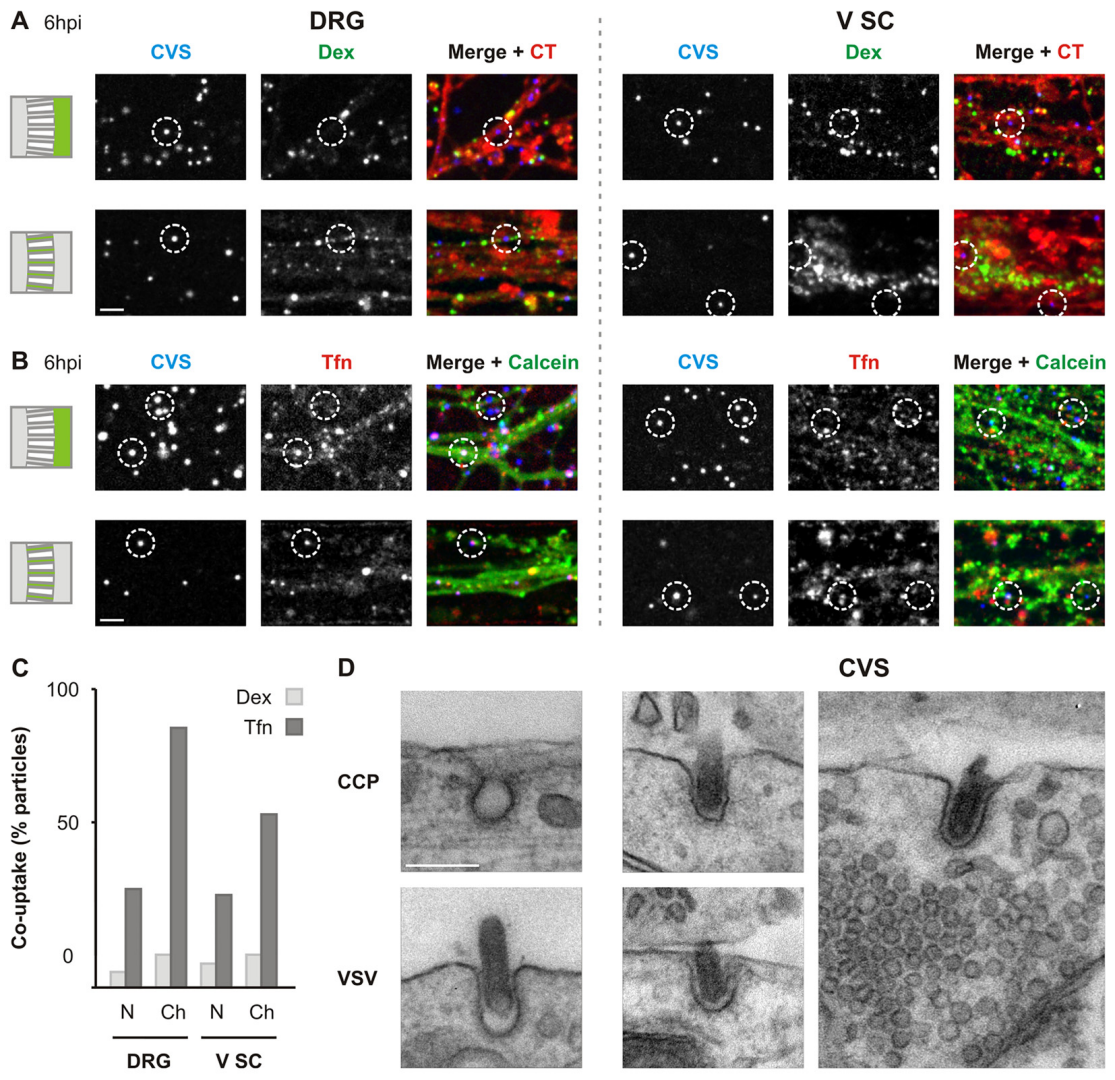
rVSV CVS G is co-packaged with transferrin during endocytosis. **A.** Confocal imaging displaying limited colocalization between incoming rVSV CVS G AF647 particles (CVS) and dextran (Dex) in DRG and V SC neurons. N compartments previously stained with CellTracker (red) were inoculated with rVSV CVS G AF647 (blue) in the presence of Dex-AF488 (green), 10,000 MW. Co-localization of virus and dextran was assessed in neurites of the N compartment (top) and microchannels (bottom) at 6 hpi as indicated. Merges combine CT, CVS and Dex signals. Dotted circles emphasize virions of interest. Scale bar = 20 μm. **B.** Confocal imaging of colocalization between incoming rVSV CVS G AF647 particles (CVS) and transferrin (Tfn) in DRG and V SC neurons. N compartments previously stained with calcein (green) were inoculated with rVSV CVS G AF647 (blue) in the presence of Tfn-AF594 (red). Co-localization of virus and Tfn was assessed as in A. Merges combine calcein, CVS and Tfn signals. Dotted circles emphasize virions of interest. Scale bar = 20 μm. **C.** Quantitation of co-uptake of virus and the indicated endocytic markers in the N compartment and microchannels. **D.** Transmission electron micrographs of endocytic structures and rVSV CVS G uptake into V SC at 2 hpi. Top left: a clathrin-coated pit (CCP). Bottom left: VSV particle undergoing clathrin-coated pit envelopment in B-SC-1 cells. Right (CVS): three representative images of endocytic structures involved in rVSV CVS G uptake into V SC neurites. Scale bar = 100 nm, and applies to all panels.

Fluorescent transferrin (Tfn) and dextran (Dex) are commonly used markers of clathrin-mediated and fluid phase endocytosis respectively. To corroborate our inhibitor studies, we next determined whether rVSV CVS G AF647 are colocalized with Tfn or Dex during entry (Fig 6A and 6B). In both neuronal populations, we observe a limited colocalization of virions with Dex in the N compartment and channel neurites (Fig 6A–6C). By contrast, a third of incoming rVSV CVS G associate with Tfn in the N compartment (Fig 6B), and this colocalization is enriched in the microchannels. This result suggests that internalization via clathrin shunts virus into long-range axoplasmic transport (Fig 6B and 6C). The transportation bias for particles originating from clathrin-coated pits is particularly pronounced within DRG neurons where 87% of virions within the channels colocalize with Tfn versus 33% in the N compartment (Fig 6C). Although fewer viral particles associate with Tfn in V SC channel neurites (Fig 6B and 6C) we note a similar enrichment from 31% in the N compartment to 58% in the channels. This observation is consistent with results showing greater sensitivity of infection to EIPA in the V SC neurons (Fig 3B). However, transmission electron micrographs of rVSV CVS G uptake in V SC displays viruses exclusively associated with endocytic structures consistent with clathrin mediated endocytosis (Fig 6D). This may suggest an off-target effect of EIPA in neurons which, by dysregulating Na^+^/H^+^ exchangers, may indirectly impact neuronal endocytic dynamics via disruption of the excitation properties of the plasma membrane [35]. Alternatively, 2hpi may be too early for the unambiguous identifications of macropinocytic structures in these cells. Furthermore, a caveat of the Tfn uptake within V SC neurons is the reported absence of transferrin receptors (TfnR) on the axons of mature motor neurons [36]. Axonal TfnR expression can be maintained by culturing of neurons in the presence of Tfn [37] and during axonal regeneration such as during outgrowth following axotomization inherent to the dissociation process [38]. Although the observation of Tfn uptake suggests that these primary cultures retain the receptor at some level, it is possible that a low expression of TfnR may result in an under-reporting of clathrin-mediated uptake in these neurons.

**Fig 7:**
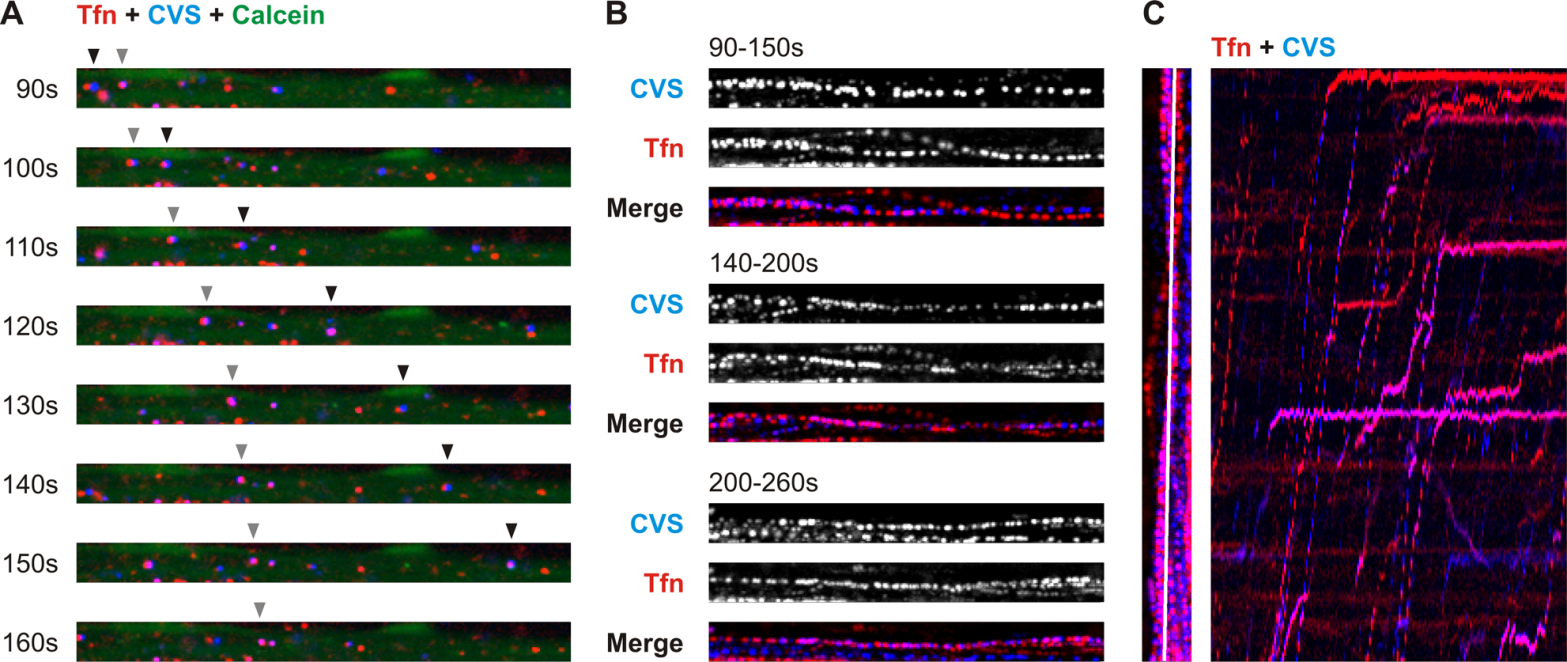
Active co-transport of rVSV CVS G and transferrin within DRG neurites. **A.** Time-lapse images from confocal live recording of co-transport of rVSV CVS G AF647 (blue) and transferrin-AF594 (Tfn, red) in calcein-stained neurites (green) within a microchannel. Timepoints indicated are matched with timestamps in S1 Video. Arrowheads track two viral particles being co-transported with Tfn. **B.** Thirty timepoints, corresponding to 60s clips from S1 Video, combined into a single image to display tracks of virus (CVS) and Tfn-positive endosomes through a microchannel from cultured DRG neurons. CVS and Tfn tracks are shown individually in greyscale, and merged. Timepoints indicated are matched with timestamps in S1 Video. **C.** Kymograph of Tfn (red) and CVS (blue) particle movement along a single plane from S1 Video. The left panel indicates in white the plane used to generate the kymograph. Each timepoint for this plane is then projected along the x axis to reveal Tfn and viral tracks intersecting the plane (right panel). Cotransport of virions and Tfn generates matching traces on the kymograph.

To ascertain that viral particles are being transported, we performed live imaging studies in DRG neurons (S1 Video and S2 Video; Fig 7). Within the channels, we recorded single viral particles in the process of long-range axoplasmic transport. Many of these were transported concomitantly with fluorescent Tfn (S1 Video).

### Fusion of rVSV CVS G occurs following transport to the soma

**Fig 8:**
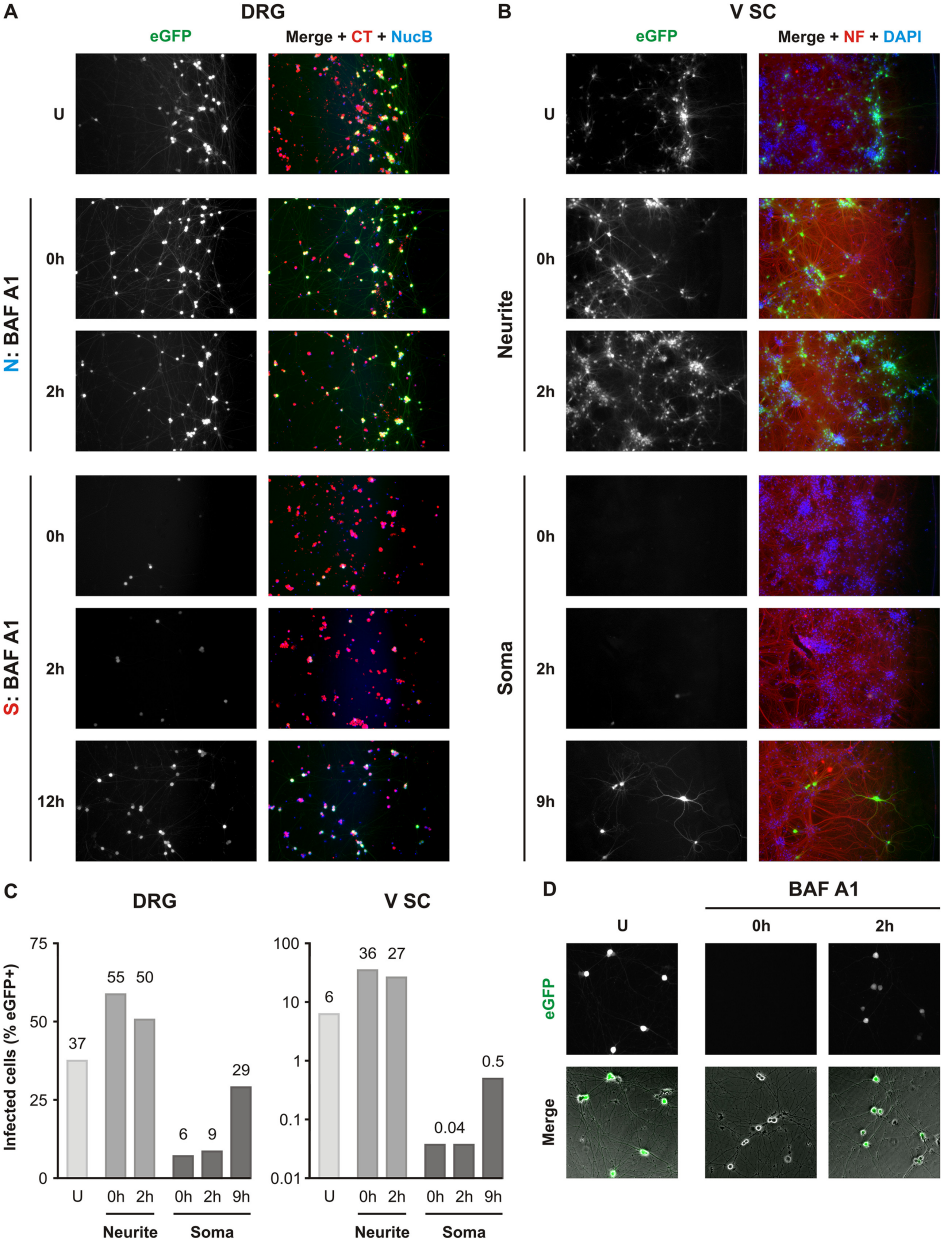
Somal endosomal acidification is required for rVSV CVS G infection. Fluorescence microscopy of compartmentalized cultures of **A.** DRG neurons or **B.** V SC neurons infected with rVSV CVS G in the N compartment in the presence or absence (U) of 1 μM bafilomycin A1 (BAF A1). BAF A1, a block of endosomal acidification, was administered to either the N or S compartment at various timepoints relative to the start of infection. In the N compartment, BAF A1 was added at 0 or 2 hpi. In the S compartment, BAF A1 was added at 0, 2, 9 or 12 hpi as indicated. Infection was assessed by expression of viral eGFP at 26hpi. DRG neurons in A. were pre-stained with CellTracker (CT, red) and NucBlue (NucB, blue) prior to the start of the experiments. In B., infected V SC neurites were fixed and permeabilized at 26hpi. V SC neurites were detected by immunofluorescence against phosphorylated neurofilament (NF, red) and nuclei were stained with DAPI (blue). **C.** Quantitation of percentage eGFP positive cells following a representative experiment in DRG (left) and V SC (right) culture. All conditions, except the untreated control, were tested in duplicate. Averages are provided above each individual bar. D. Fluorescence microscopy of non-compartmentalized DRG cultures infected with rVSV CVS G and treated with BAF A1 at 0 or 2 hpi. U indicates untreated controls. Presence of the infection marker, eGFP (green), was assessed at 8 hpi. Merged images combine the eGFP signal and phase microscopy of the DRG neurons in culture.

Within highly polarized neurons, fusion can occur locally at the site of uptake or following endosomal transport at intermediate locations along the axon or at the cell body. Live imaging data of cotransport with Tfn corroborates previously published findings that RABV virions are sorted into a long-range vesicular transport route with delayed acidification and release at the cell body [21–23]. To investigate the role of delayed fusion on productive infection we administered Bafilomycin A1 (BAF A1), an inhibitor of vesicular H^+^ ion pumps, to the N or S compartment to respectively block localized or delayed fusion. We monitored the impact of this selective treatment on infection (Fig 8A–8C). Cells whose neurites were treated with BAF A1 displayed widespread infection in the S compartment despite presence of the inhibitor (Fig 8A and 8B). Under these conditions, we also observed enhanced viral spread relative to untreated counterparts in both DRG and V SC neurons. In contrast, somal treatment with BAF A1 resulted in robust inhibition of infection (Fig 8A–8C). This effect depended on administration of the drug early in infection: we observed lesser inhibition following treatment at 9–12 hpi. At this point incoming viruses from the N compartment have likely already fused at the cell body. We excluded potential cytotoxicity or off-target effects on the viral life cycle by assessing the effect of BAF A1 in non-compartmentalized culture. Post-entry administration at 2 hpi did not interfere with infection (Fig 8D).

**Fig 9:**
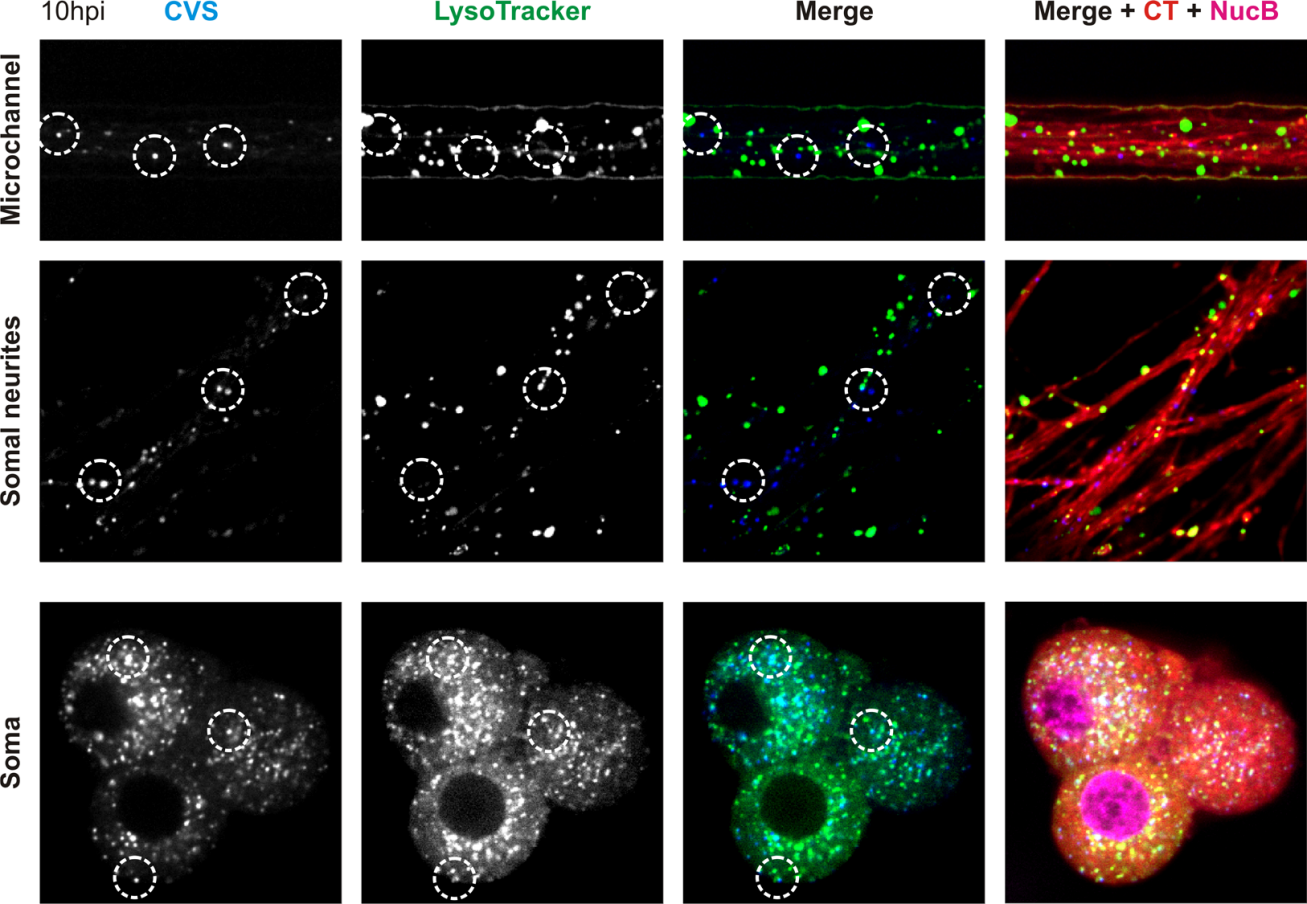
rVSV CVS G viruses are exposed to acidic pH within the cell body of DRG neurons. Confocal microscopy of rVSV CVS G AF647 virions (blue) and the marker for acidified endosomes, LysoTracker (green) during uptake into DRG neurons pre-stained with CellTracker (CT) and NucBlue (NucB). Virus and LysoTracker were administered to the N compartment and localization of virions within acidified endosomes was assayed at 10hpi in the microchannel neurites, somal neurites and cell bodies. Dotted circles emphasize virions of interest.

We corroborated our inhibitor experiments by demonstrating that incoming fluorescently-labeled viral particles colocalize with LysoTracker in the cell bodies of DRG neurons (Fig 9). LysoTracker accumulates in acidified endosomes where the luminal pH is low enough to trigger fusion. No colocalization was observed between LysoTracker and virions within microchannel neurites or neurites proximal to the somatodendritic compartment (Fig 9; S3 Video). We did not investigate colocalization of virions with LysoTracker at the growth cones or in distal neurites. Although we were unable to conclusively determine whether viruses enter acidified organelles prior to arrival in the cell body, the presence of acidified endosomes within the neurites is consistent with the possibility of fusion at earlier timepoints (Fig 9). Together, these results demonstrate that the majority of incoming virions are transported within endosomes to the cell body where acidification occurs. Fusion at the cell body is then a prerequisite for efficient infection.

## Discussion

**Fig 10:**
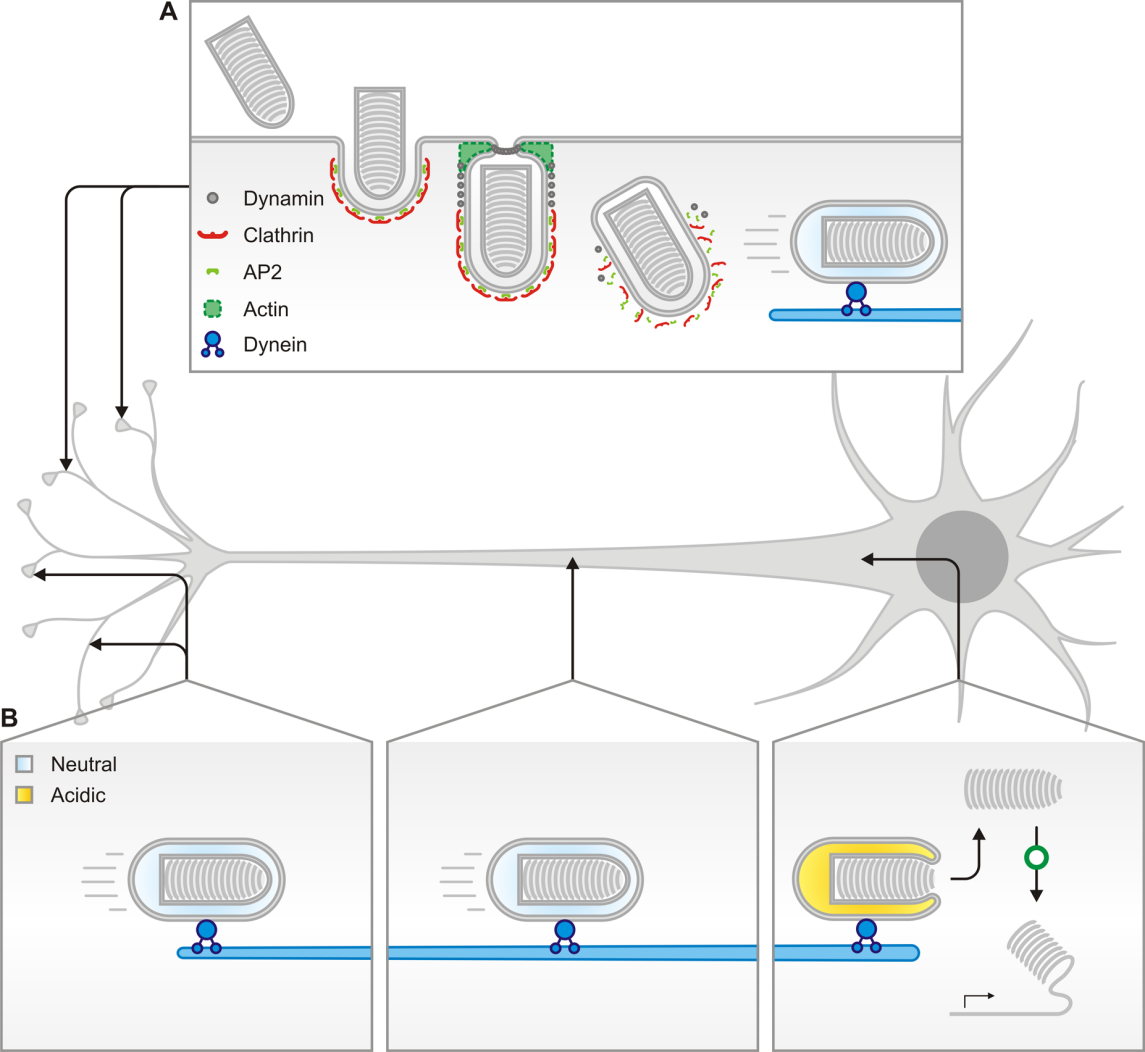
Model of the predominant RABV G-dependent entry mechanism into neurons of the peripheral nervous system. Rabies G association with neuronal receptors at the synapse or neurite membrane results in clathrin-mediated endocytosis and transport via microtubules to the somatodendritic compartment. **A.** Rhabdoviruses are internalized within endosomes only partially coated with clathrin, which require actin to complete envelopment and scission from the plasma membrane. **B.** Following internalization, early endosomes containing virus engage the motors and travel retrogradely through the axon. Viruses are transported intact within endosomes from the neuronal synapse. Endosomes acidify upon arrival at the soma releasing RNPs and leading to productive infection.

Here we provide evidence for a model of RABV entry into peripheral neurons that begins with clathrin- and dynamin-mediated uptake at the plasma membrane (Fig 10A). Endocytosed viruses are then transported intact and within endosomes from the distal neurites to the site of fusion at the cell body (Fig 10B). Furthermore, we show that somal fusion is required for efficient infection. This work extends the current understanding of RABV uptake by identifying the predominant internalization mechanism at the plasma membrane of two neuronal populations involved in early neuroinvasion *in vivo*. In addition, by combining single particle imaging and infectivity studies, we correlate single virion behavior with productive infection.

### Viral fusion occurs following long-range transport to the soma

In this study, we provide corroborating evidence of long-range axoplasmic transport of rhabdoviruses incorporating RABV G. Co-transport of virions with Tfn is consistent with trafficking within endosomes. Furthermore, exclusive sensitivity of infection to disruption of endosomal acidification in the S compartment demonstrates that viral fusion events leading to productive infection occur in the vicinity of the soma. Previous studies have reported long-range endosomal transport of intact vaccine strain RABV virions within differentiated neuroblastoma cells and DRG neurons [21,23]. Additionally, lentiviral vectors pseudotyped with G from the attenuated CVS-B2C strain have been tracked during retrograde endosomal transport in compartmentalized motor neuron culture [22]. We extend those earlier observations to the pathogenic CVS-11 G, and demonstrate that subsequent efficient establishment of infection is facilitated by pH-dependent fusion at the perikaryon.

Previous work with the SAD vaccine strain of RABV provided evidence for transport within acidified endosomes [23]. This is in contrast to our observation that rVSV CVS G is exposed to acidic endosomal pH primarily at the cell body of neurons (Fig 9; S3 Video), with a minority of particles in acidified endosomes in the microchannel neurites. Vaccine strains of RABV are characterized by their reduced neurotropism and neuroinvasiveness, which are dictated by their respective glycoproteins and may account for the difference between our observations. The site of imaging might also be a contributory factor in the apparent difference because studies of acidified endosome distribution in neurons suggest a high density of these structures in areas proximal to the growth cone, but a significant reduction at intermediate locations on the axon [39].

The increase in infection and spread following inhibition of endosomal acidification at the neurites in both DRG and V SC neurons suggests that some virus-containing endosomes engaging long-range axoplasmic transport may undergo acidification before delivery to the soma (Fig 8). The presence of RABV in acidified organelles at the neuronal termini or within distal neurites has been previously suggested based on colocalization between RABV antigens and LysoTracker at NMJs [20], and cotransport of vaccine strain RABV and LysoTracker within DRG neurons [23]. Studies of tetanus neurotoxin trafficking suggest that sorting of certain cargoes into axonal carriers involves passage through intermediate acidified endosomes [40]. Although we did not independently verify the presence of infectious particles within acidified organelles in the distal neurites and growth cones, we cannot exclude that a similar sorting step occurs during transportation of RABV virions. If rhabdoviral particles traffic through analogous structures, exposure to the acidified environment may result in early fusion of a subset of incoming virions. Our results suggest that such early triggering of fusion leads to a delay or possibly abrogation of infection. Accordingly, if this early acidification event is circumvented–for example by BAF A1 inhibition of the vacuolar ATPase–viruses that would otherwise have fused prematurely now proceed along the transportation route and are subject to subsequent acidification events leading to infection. Although this observation is provocative it is important to emphasize that here we are studying infection initiated by delivery of the VSV RNP core and we cannot ignore the possibility of a RABV specific RNP transit to the cell body. The reported interaction between RABV P, and dynein LC8 [41,42] could facilitate RNP engagement of microtubules for retrograde transport. Mutation of the motif in P responsible for this interaction does not, however, abolish neuroinvasiveness of RABV *in vivo* [43,44] suggesting that direct transport of RNPs is not the primary mechanism of RABV axoplasmic transport.

### Evidence for rhabdoviral uptake into partially-coated clathrin pits in neurons

Previous studies investigated RABV uptake into non-neuronal [18,19], neuroblastoma [17] or hippocampal neurons [16]. Our work extends these studies into DRG and V SC neurons, the first populations of neurons invaded by rabies *in vivo*. We identify clathrin-mediated endocytosis (CME) as the primary mechanism of productive RABV uptake. We base our conclusion on three observations: (i) susceptibility of infection and single particle uptake to disruption of dynamin; (ii) co-packaging and -transport of incoming virions with transferrin; and (iii) detection of rhabdoviral particles within coated pits by electron microscopy. Because co-transport with transferrin was assessed during long-range axoplasmic transport, we cannot exclude that rhabdovirus-containing endosomes fuse or coalesce with Tfn-positive endosomes following internalization at the plasma membrane. However, transmission electron micrographs of uptake into V SC neurons provide direct evidence for clathrin-dependent uptake as viruses associated with coated endocytic structures that resemble clathrin-coated pits.

Ultrastructural data from transmission electron micrographs also allows us to infer additional structural and mechanistic similarities between rhabdovirus-containing endosomes in neuronal and epithelial cells. The clathrin-coated pits share the elongated profile characteristic of their incompletely coated counterparts observed in BS-C-1 cells for both VSV and RABV (Fig 6D) [19,45,46]. It is likely, therefore, that actin polymerization is a requirement for completion of envelopment and scission also from the neuronal plasma membrane.

### RABV G-mediated uptake of rhabdoviruses into two neuronal populations

Sensory DRG neurons and motor neurons are both susceptible to RABV infection in the host. Due to the morphological and functional differences between these neuronal populations, we explored the possibility of non-identical uptake mechanisms for RABV based on the neuronal subtype. Our infectivity experiments using pharmacological perturbation of endocytic processes reveal that productive infection in either neuronal population is dynamin-dependent. Single particle experiments further implicate clathrin-mediated uptake as a major route of RABV endocytosis in both cell types. Accordingly, 90% of particles in DRG microchannel neurites, and 55% in V SC neurites are co-packaged with transferrin.

We also identified some differences in uptake between the two neuronal populations. In V SC culture, a significant fraction of incoming particles do not colocalize with transferrin, or fluorescent dextran. This suggests that a fraction of particles enter in a manner independent of clathrin or macropinocytic uptake mechanisms. Such differences in uptake appear to be cell-type dependent and may be dictated by differential engagement of particular receptors. Lentiviral vectors expressing CVS-B2C G undergoing axoplasmic transport in motor neurons were found to colocalize with all three known RABV receptors: p75 neurotrophin receptor, neural cell adhesion molecule, and nicotinic acetylcholine receptor [22]. Studies of uptake of putative rabies receptors following engagement with endogenous ligands, crosslinking antibodies or toxins indicate that p75^NTR^, NCAM and nAChR internalize by different cellular mechanisms. p75^NTR^ bound to neurotropic factors internalizes via clathrin-mediated endocytosis [47–49]. Antibody crosslinking of NCAM induces its internalization primarily by clathrin-mediated endocytosis with caveolae playing a secondary role [48,49]. Finally, nAChR uptake into filamentous invaginations from the plasma membrane is clathrin-independent [50,51]. These observations raise the possibility that RABV interaction with specific receptors may contribute to the endocytic sorting of the virus. It will therefore be of interest to identify which receptors are internalized at the plasma membrane in complex with RABV and to relate these interactions to establishment of infection in these two neuronal populations.

## Materials and Methods

### Fabrication of microfluidic devices

Device masters were manufactured by two-layer soft photolithography onto 3 inch mechanical grade silicon wafers for spin coating (UW3MEC, University Wafers) utilizing established methods [52]. Two negative photoresists were used: SU-8 2002.5 (MicroChem) for the 3 μm microchannel layer followed by SU-8 2050 (MicroChem) for the 100 μm culturing compartment layer. Photoresist was patterned by UV-crosslinking through custom 20,000 dpi transparency masks (CAD/Art Services), processed and cured according to supplier’s instructions. Following a final hard cure at 150°C for 15 min, masters were treated with (tridecafluoro-1,1,2,2-tetrahydrooctyl)trichlorosilane for 45 min to facilitate removal of cured polydimethylsiloxane (PDMS) following moulding. Devices were cast by applying a 10:1 prepolymer:curing agent mixture of Sylgard 184 (Dow Corning) to the master and curing at 65°C for a minimum of 1 h. After curing and release from the master, PDMS devices were cut, and wells punched out with round biopsy punches. We irreversibly bonded devices to acid cleaned glass coverslips by oxygen plasma bonding in a 500-II Plasma Etcher (Technics). Bonded devices were sterilized under UV in a biosafety cabinet for 10 minutes prior to consecutive overnight coatings with 300 μg ml^-1^ poly-D-lysine (P7886, Sigma-Aldrich) dissolved in 2X borate buffer solution (28341, Thermo Scientific) and 10 μg ml^-1^ laminin (L2020, Sigma-Aldrich) in sterile water.

### Ethics statement

Pregnant Spraque-Dawley rats (Taconic) were a kind gift from C. Cepko. All animal work included in this study was approved by the Harvard Medical Area Standing Committee on Animals under protocol 428-R98 of the Institutional Animal Care and Use Committee (IACUC) of Harvard Medical School. Animals were housed and handled in accordance with the *Guide for the Care and Use of Laboratory Animals*. Euthanasia of pregnant Sprague-Dawley rats was performed by controlled exposure to carbon dioxide from compressed gas cylinders such that suffering and distress was minimized. Whenever possible, animals were kept in their housing cages during the procedure. Non-responsive animals were further subjected to cervical dislocation to exclude any possibility of accidental revival. Only once this sequence of procedures was completed did we remove the uterus and embryos from the carcass. E15 embryos were euthanized by removal from the amniotic sac and subsequent decapitation.

### Neuronal culture

Neuronal tissues were dissected from E14.5-E15.5 embryonic Sprague-Dawley rats (Taconic). Dorsal root ganglia (DRG) were dissected, dissociated by trypsinization and cultured in Neurobasal media (Gibco) supplemented with B27 (1:50; 17504–044, Gibco), β-nerve growth factor (100 ng ml^-1^; 450–01, Peprotech), 5% fetal bovine serum (FBS, Tissue Culture Biologicals), 2 mM glutamine (101806, MP Biochemicals), 25 mM HEPES (0511, AMRESCO) pH 7.4 and 25 μg ml^-1^ β-D-arabinofuranoside (AraC; C1768, Sigma-Aldrich) [53]. Ventral spinal cord neurons were dissected by adapting the strategy outlined for the harvest and culture of dorsal spinal cord commissural neurons [54]. Instead of harvesting the dorsal portion of the spinal cord, we retained the ventral portion for our cultures. Dissociation of the neurons was also carried out as detailed with the exclusion of the Opti-Prep purification step. V SC neurons were cultured in Neurobasal media (Gibco) supplemented as outlined by Leach et al. [55]. AraC treatment for selective kill-off of dividing non-neuronal cells was included and maintained in DRG media from first plating. For V SC culture AraC was applied after 48 h in culture.

For preparation of compartmentalized cultures, we seeded dissociated neurons into the S compartment: 1.5 × 10^5^ DRG or 1 × 10^5^ V SC neurons were dispensed per device. A half-media swap was performed for both DRG and V SC culture following 2–3 days in culture; at this point, 25 μg ml^-1^ AraC (Sigma-Aldrich) was administered to the V SC cultures and maintained. Subsequently, media was periodically supplemented to counteract evaporation in culture. Experimental infections were carried out once adequate neurite outgrowth was observed in the N compartment: typically, from day 7 in culture for DRG and day 10 for V SC neurons. Devices were discarded following 12 days in culture.

### Cells and virus preparation

Neurons, mouse neuroblastoma Neuro-2a cells (N2a; ATCC CCL-131; American Type Culture Collection, Manassas, VA), baby hamster kidney BSR T7/5 cells (gift of U.J. Buchholz) [56], and African green monkey kidney BS-C-1 cells (ATCC CCL-26; American Type Culture Collection, Manassas, VA) were maintained at 37°C and 5% CO_2_. Non-neuronal cells were cultured in Dulbecco’s modified Eagle medium (DMEM; Corning) supplemented 10% fetal bovine serum (Tissue Culture Biologicals). N2a media was further supplemented with 2mM glutamine (Sigma) and 25mM HEPES pH 7.4. rVSV eGFP and rVSV eGFP SAD B19 G were amplified, purified and maintained as previously described [45,46]. rVSV eGFP CVS G (rVSV CVS G) was generated by insertion of the CVS-11 glycoprotein coding region into MluI and NotI restriction sites in a modified rVSV eGFP ΔG backbone [57–59]. A pUC57 plasmid containing the cDNA of CVS-11 G (Genbank: GQ918139.1) with flanking MluI and NotI sites was commercially synthesized by GenScript. A P0 stock of the virus was recovered by standard methods in BSR T7/5 monolayers [45]. Individual viral clones from the P0 stock were isolated by fluorescent focus assay in N2a culture, and further amplified in N2a monolayers.

rVSV CVS G stocks were passaged and expanded in N2a monolayers. Infections were carried out according to standard technique [45]. At 24hpi supernatant and infected N2a cells were collected and subjected to 2 min sonication in a Branson 1510 ultrasonic cleaner (Branson, Richmond, VA) followed by 30 s vortex to release cell bound viruses. Cell debris was pelleted by centrifugation and the resultant virus supernatant purified by ultracentrifugation. Virus pellets were resuspended overnight in phosphate buffered saline (PBS) + 25 mM HEPES pH 7.4 + 50 mM EDTA. We sonicated the virus resuspension for an additional 2 min, followed by 30s vortex, immediately prior to a final gradient purification over a 15%-45% (wt/vol) sucrose gradient in PBS as previously described [45]. Viral titers were determined by fluorescent focus assay in N2a monolayers.

We used established methods to label gradient-purified viral particles with 40 μg ml^−1^ Alexa Fluor (AF)-conjugated succinyl esters (Molecular Probes, Invitrogen) [45]. Titration of mock- or AF647-labeled virus preparations showed that dye conjugation had a negligible effect on infectivity: mock-labeled rVSV CVS G had a titer of 2.1 x 10^10^ ffu/mL compared to 1.5 10^10^ ffu/mL titer of the AF647-labeled virus.

### Protein composition of purified virions

Viral proteins of gradient purified virions were separated by SDS-PAGE in a 10% polyacrylamide (wt/vol) and 0.13% (wt/vol) bis-acrylamide gel. Protein bands were visualized with SimplyBlue SafeStain according to manufacturer’s instructions. Viral protein amounts relative to N protein were determined using ImageJ (U.S. National Institutes of Health, Bethesda, Maryland; http://rsb.info.nih.gov/ij/).

### Live staining of neurons in compartmentalized culture

Neuronal cytoplasms in the N and S compartments were stained with calcein (diluted 1:1000; C3099, Molecular Probes) or CellTracker (diluted 1:500; C34552 Molecular Probes), and nuclei with NucBlue Live Cell Stain (diluted 1:50; R37605, Molecular Probes) in Neurobasal for 30 min prior to inoculation with virus. Stains were washed once with Neurobasal following removal, prior to infection or imaging by direct fluorescent microscopy.

### Inhibitors

The following chemicals were administered at the listed concentrations: 0.1 μM bafilomycin A1 (BAF A1; 196000, Calbiochem, EMD Chemicals); 150 μM dynasore (Sigma-Aldrich); and 25 μM 5-(*N*-ethyl-*N*-isopropyl)amiloride (EIPA; A3085, Sigma-Aldrich).

### Infections in compartmentalized culture

Infections were carried out exclusively in the N compartment. For N-compartment inhibitor treatment, culturing media in the S compartment was supplemented to replace evaporated liquid volume, and maintained throughout the experiment. For BAF A1 experiments where inhibitor treatment was also carried out in the S compartment, S compartment media was replaced with Neurobasal alone or BAF A1 diluted in Neurobasal. Neuronal culturing media in the N compartment was replaced with 30 μL of inoculation media. Inoculation media consisted of Neurobasal media alone or with inhibitor as indicated. 10^6^ fluorescent foci forming units of virus were administered to the N compartment in 25 μL of inoculation media. Liquid volume differential between the S compartment and N compartment was optimized to eliminate diffusion of molecules across the channels. Inoculum was maintained for 2 h and then replaced with 55 μL treated or untreated Neurobasal media. For BAF A1 experiments where inhibitor was administered at 9–12 hpi, an additional media swap was carried out. Expression of eGFP was assessed at 26 hpi either by direct live fluorescence microscopy for DRG neurons or following fixation and immunofluorescence for V SC neurons. This end point was determined by monitoring appearance of eGFP and selecting a timepoint at which robust eGFP expression first occurs. In addition, we verified infection of neurons solely in the vicinity of the microchannel openings, consistent with primary infection without spread to secondary infection sites.

### Co-uptake of RABV with fluorescent transferrin, dextran and LysoTracker

For all co-uptake experiments neurons were first prestained with calcein or CellTracker as indicated. Infections were carried out, as described, in the N compartment with inocula containing Tfn conjugated to AF594 (50 μg ml^-1^; Molecular Probes), AF488-conjugated Dextran (MW 10 000, 1 μg ml^-1^; Molecular Probes) or LysoTracker DND-26 (75 nM; Molecular Probes). Tfn and Dextran results were analyzed in both fixed and live samples. All LysoTracker images were collected by live confocal microscopy. For fixed experiments, N compartments were washed twice at 2 or 5 hpi with Neurobasal, and fixed with 2% (wt/vol) paraformaldehyde in PBS + 5% (wt/vol) sucrose. For live imaging, uptake experiments were carried out in devices bonded to FluoroDish glass bottomed culture dishes (FD35-100, World Precision Instruments, Inc.) and imaged by high resolution spinning disk confocal microscopy.

### Immunofluorescence

Neurons were fixed with 2% (wt/vol) paraformaldehyde in PBS + 5% (wt/vol) sucrose. Cell membranes were permeabilized with 0.2% Triton-X in PBS. Cells were consecutively stained with mouse monoclonal antibody against phosphorylated neurofilament H, SMI-31 (1:1000; NE1022, Calbiochem), and AF-conjugated anti-mouse secondary antibody (Molecular Probes, Invitrogen). When indicated, neuronal cells were detected by staining against Neuronal Nuclei (NeuN) with rabbit polyclonal antibody (1:500; ab104225, Abcam) and AF-conjugated anti-rabbit secondary antibody (Molecular Probes). Nuclei were stained with DAPI (1:10,000; Molecular Probes). Devices processed for immunofluorescence underwent a final wash with PBS and were imaged in solution. Non-compartmentalized neurons, cultured on coverslips, were mounted onto slides with ProLong Diamond (Molecular Probes).

### Fluorescence microscopy

Devices were illuminated with a Mercury-100W mercury lamp (Chu Technical Corporation) and imaged using a Nikon Eclipse TE300 inverted microscope, outfitted with 4× Plan Fluor, 10× and 20× Plan Fluor objective lenses (Nikon). Images were collected using a SPOT RT Monochrome camera (Spot Imaging Solutions, Diagnostic Instruments Inc.) and recorded with the manufacturer’s Spot 3.5 Advanced software.

### Spinning disk confocal microscopy

Devices were imaged using a Marianas system (Intelligent Imaging Innovations) based on a Zeiss observer microscope (Carl Zeiss MicroImaging) outfitted with a CSU-22 spinning-disk confocal unit (Yokogawa Electric Corporation) and a 63× (Plan-Apochromat, NA 1.4; Carl Zeiss Microimaging) objective lens. Excitation wavelengths were 491 nm for AF488, 561 nm for AF594, and 660 nm for AF647. For three-dimensional acquisitions, the vertical position was manipulated in 0.3 μm increments using a PZ-2000 automated stage (Applied Scientific Instrumentation). Live imaging experiments were carried out on a temperature controlled sample holder (20/20 Technology Inc.; Wilmington, NC) maintained at 37°C and 5% CO_2_. Images were collected using a Photometrics Cascade II electron multiplication camera (Photometrics). SlideBook 5.0 (Intelligent Imaging Innovations) was used to command the hardware devices, and visualize and export the acquired data. Subsequent image manipulation was conducted using ImageJ (U.S. National Institutes of Health, http://rsb.info.nih.gov/ij/).

### Transmission electron microscopy

To visualize viral morphology, we deposited gradient purified rVSV CVS G virions onto carbon-coated copper grids and stained them with 2% phosphotungstic acid (wt/vol) in H_2_O (pH 7.5). To visualize viral uptake, we inoculated V SC neurons cultured on Aclar with rVSV CVS G at a multiplicity of infection (MOI) exceeding 1,000 for 2 h at 37°C. rVSV uptake samples were prepared by inoculating BS-C-1 cells with rVSV at an MOI of 1,000 for 15 min at 37°C. Samples were fixed and processed for ultrathin sectioning as previously described [45,60]. Virus particles and ultrathin sections of cells were viewed using a Tecnai G^2^ Spirit BioTWIN transmission electron microscope (FEI).

## Supporting Information

S1 Fig Fluorescence microscopy of calcein-stained, compartmentalized DRG and V SC neurons demonstrating continuity of neurites through the microchannels. Scale bars = 100 μm. Insets show enlargements of representative microchannels containing intact neurites.
(TIF)

S1 Video Retrograde cotransport of rVSV CVS G AF647 with fluorescent transferrin (Tfn). Time-lapse imaging of microchannel neurites of a compartmentalized culture of DRG neurons stained with calcein (green). AF647-labeled rVSV RABV G particles (blue) and Tfn-AF594 (red) are imaged by high resolution live confocal microscopy at 2s intervals at 3hpi. Retrograde axoplasmic transport is directed from bottom to top.
(AVI)

S2 Video Time-lapse imaging of particles tracked in Fig 7. Compartmentalized culture of DRG neurons stained with calcein was inoculated with AF647-labeled rVSV CVS G particles (blue), and imaged by high resolution live confocal microscopy at 2s intervals at 3 hpi.
(AVI)

S3 Video rVSV CVS G AF647 particles undergo retrograde transport within neutral endosomes. Time-lapse imaging of microchannel neurites of a compartmentalized culture of DRG neurons stained with CellTracker (red). AF647-labeled rVSV RABV G particles (blue) and LysoTracker (green) are imaged by high resolution live confocal microscopy at 3s intervals at 1hpi. Retrograde axoplasmic transport is directed from bottom to top.
(AVI)
