# Supplementary figures and images for: Rabies Internalizes into Primary Peripheral Neurons via Clathrin Coated Pits and Requires Fusion at the Cell Body
Source: PLoS Pathog. 2016 Jul 27;12(7):e1005753. doi: 10.1371/journal.ppat.1005753 (PMC4963122; doi:10.1371/journal.ppat.1005753)

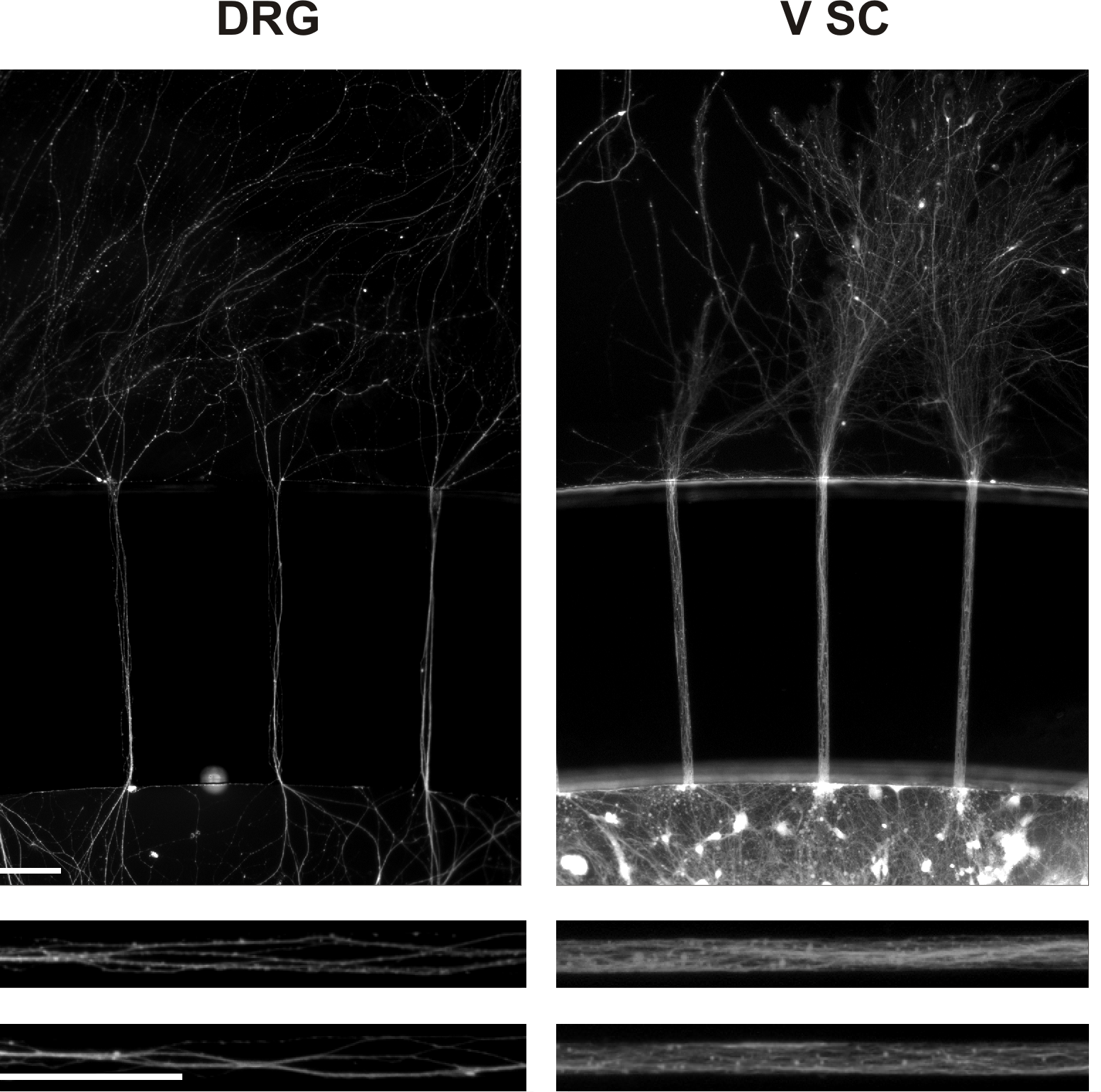

Supplement: S1 Fig — Scale bars = 100 μm. Insets show enlargements of representative microchannels containing intact neurites. (TIF) [file ppat.1005753.s001.tif]
